# Survival and prognosis of young adults with gastric cancer

**DOI:** 10.6061/clinics/2018/e651s

**Published:** 2018-09-11

**Authors:** Marina Candido Visontai Cormedi, Maria Lucia Hirata Katayama, Rodrigo Santa Cruz Guindalini, Sheila Friedrich Faraj, Maria Aparecida Azevedo Koike Folgueira

**Affiliations:** I Departamento de Radiologia e Oncologia, Instituto do Cancer do Estado de Sao Paulo (ICESP), Faculdade de Medicina FMUSP, Universidade de Sao Paulo, Sao Paulo, SP, BR; II Departamento de Patologia, Instituto do Câncer do Estado de Sao Paulo (ICESP), Faculdade de Medicina FMUSP, Universidade de Sao Paulo, Sao Paulo, SP, BR

**Keywords:** Stomach Neoplasms, Survival, Young Adult

## Abstract

**OBJECTIVES:**

Survival data for young adults (YA) with gastric cancer is conflicting and scarce in Brazil. The aim of this study was to compare the clinicopathological factors and survival rates of younger and older patients with gastric cancer.

**METHODS:**

Hospital registries for 294 gastric cancer patients from a reference cancer hospital in São Paulo, Brazil, were consulted for the retrieval of clinicopathological information and follow-up time. Patients were placed into the following groups: YA (≤40 years; N=71), older adult (OA: 41 to 65 years; N=129) and elderly (E: ≥66 years; N=94). Differences were assessed through Pearson's χ^2^ test, Kaplan-Meier analysis, Log rank test and Cox regression.

**RESULTS:**

More YA were diagnosed with advanced disease (clinical stage III/IV: 86.7% YA, 69.9% OA, and 67% E); however, fewer E patients underwent surgery (64.3% YA, 72.7% OA, and 52.4% E). The median overall survival among all patients was 16 months, and the overall survival rate was not significantly different among the age groups (*p*=0.129). There were no significant differences in the disease-free survival rate. Metastatic disease at diagnosis (HR=4.84; *p*<0.01) was associated with an increased hazard of death for YA.

**CONCLUSION:**

Overall survival was similar among age groups. Metastatic disease at diagnosis was the only factor associated with a poorer prognosis in YA. These results suggest that younger patients deserve special attention regarding the detection of early stage disease.

## INTRODUCTION

Gastric cancer is the fifth most common cancer worldwide and is one of the deadliest types, accounting for 723,000 deaths worldwide in 2012 1. Although global gastric cancer incidence and mortality rates have been declining worldwide, an increased or flattened incidence rate has been observed in young patients 2,3. In a study performed in the USA, the gastric cancer incidence among white adults aged 25 to 39 years increased from 0.27 per 100,000 person-years in 2002 to 0.45 in 2006 2. However, there are conflicting findings with respect to the survival of young patients. While some studies have reported lower survival rates in this age group 4,5, others have reported a better prognosis compared to older individuals 6,7, and some still have described no differences in survival based on age 8,9.

In Brazil, 20,000 new gastric cancer cases were expected in 2016 10. This type of cancer remains the second leading cause of cancer-related death in our country, accounting for 14,182 deaths in 2013 11. Although only scarce data are available in Brazil, stomach cancer incidence and mortality trends appear to be declining, similar to what is observed in other countries 12-14. In São Paulo state, which comprises almost 22% of the Brazilian population, there is evidence that the stomach cancer age-specific incidence rate is falling in all age groups, except in young female adults, in whom it apparently has attained a steady state 15. Regarding mortality, a recent study reported that mortality rates have markedly increased in patients aged <50 years compared to older patients over a 5-year period 13.

Recently, there has been increasing interest in characterizing cancers that affect adolescents and young adults (YAs). According to the definition of the NCI Progress Review Group, this age group comprises individuals aged 15-39 years 16; however, cut-off limits considered in various types of cancer range from 40 to 50 years 17. In adolescents and YAs, cancer is associated with high physical and psychological morbidity. Nevertheless, only a few studies have focused on cancer in this specific population, and many questions remain regarding carcinogenesis, prognosis and treatment.

Previous studies demonstrated that early age of gastric cancer onset may be associated with some clinicopathological particularities. Females are more frequent among younger patients (in all-age stomach cancer, the male to female ratio is 2:1), and diffuse and undifferentiated histologic types are more frequently diagnosed in younger patients than in older people (in whom the intestinal type is more frequent) 4-7,18. Moreover, young people usually present with disease at a more advanced stage 4-6.

In Brazil, a study investigating differences between younger and older gastric cancer patients included 39 individuals aged <45 years and found a higher incidence of diffuse-type gastric cancer and a female preponderance in the younger age group 19. Our research group is also involved in evaluating lifestyle factors that may be associated with gastric cancer. Previously, we have found that compared to older patients, those aged <55 years usually have greater ingestion of red and processed meat, both considered risk factors for gastric cancer development (manuscript submitted for publication). To the best of our knowledge, no study has explored survival and clinicopathological features simultaneously according to age in Brazil.

Therefore, gastric cancer is still a major cause of concern in our country, and only scarce data are available regarding clinical characteristics and survival for the subgroup of YAs. Thus, our aim was to compare the clinicopathological features and survival of younger and older patients with gastric cancer in a reference cancer hospital in São Paulo, Brazil. These data may contribute to distinguishing whether YAs have special needs requiring specific care.

## MATERIALS AND METHODS

### Patients

This observational retrospective study was conducted at Instituto do Câncer do Estado de São Paulo (ICESP) in São Paulo city, Brazil. São Paulo is the largest city in Brazil, and its metropolitan area has more than 20 million people 20. ICESP draws from the Brazilian public health network (SUS, Sistema Único de Saúde), which is responsible for delivering health care to nearly ¾ of our population, representing approximately 150 million people 21. ICESP is the largest reference hospital for cancer treatment in Latin America and delivers care to approximately 10% of São Paulo state gastric cancer patients referred from primary and secondary care 22. This study was approved by the Institutional Ethics Committee (Comitê de Ética em Pesquisa da Faculdade de Medicina da Universidade de São Paulo, protocol n° 48390115.2.0000.0065).

The inclusion criteria were as follows: 1) diagnosis of gastric carcinoma according to the World Health Organization International Classification of Diseases (WHO - ICD 16) and 2) registry date at ICESP between January 2011 and December 2013. The exclusion criteria were 1) gastric carcinoma diagnosis before 2010 or after 2013 and 2) diagnosis of malignant neoplasia of the stomach not otherwise specified (ICD 16.9). Because age limits for both YAs and elderly (E) people are not internationally defined, cut-offs were set at 40 years for YAs, as reported in similar previous studies 4,6,8,9, and at 65 years for E patients, as is commonly used in Brazil.

During the study period, 1209 patients were registered at our hospital for investigation or treatment of gastric cancer, and all of them were assessed for eligibility. Of these, the hospital registries of 84 patients (6.9%) who were 40 years old or younger were reviewed. From the remaining 1125 patients, we randomly selected 3 controls for each patient using the Microsoft Excel™ 2016 function “RANDBETWEEN” and thus included 252 older patients for data collection and analysis.

**Figure 1:**
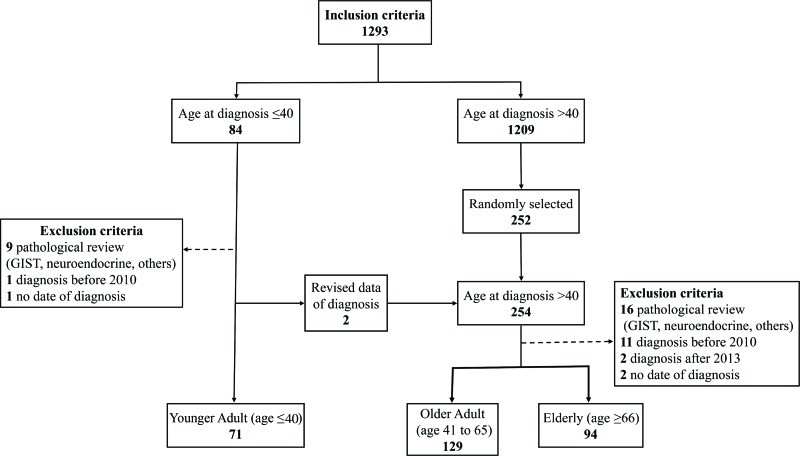
Flowchart of patient selection.

After reviewing the patients' files, eleven young patients and 31 older patients were excluded based on the exclusion criteria. Moreover, two young patients were reclassified to the older group after correcting their date at diagnosis. Finally, patients diagnosed at 40 years or younger were considered YA (N=71), and the remaining 223 patients were divided into older adult (OA; N=129), aged 41 to 65 years old, and E (N=94), aged 66 or older. Figure 1 summarizes patient selection.

Clinicopathological information, including sex, age at diagnosis, diagnostic method, histological type according to Lauren classification 23, TNM classification 24, date of recurrence, date of death and whether the patient underwent curative surgery and received chemotherapy, were retrieved from the hospital registry. Lifestyle factors were not evaluated because such data were missing for most patients. For overall survival, follow-up time was calculated from the date of diagnosis to the date of death or of last hospital registry until December 2016. Diagnostic methods were as follows: 1) stomach biopsy before surgery (86.4% of cases); 2) pathology report of gastric surgery (8.8%); 3) biopsy of metastatic sites or fine needle aspiration of primary tumor site (1.7%); 4) computed tomography of inoperable metastatic disease (2.6%); and 5) not reported (0.3%). Histological classification was reviewed by a pathologist before analysis.

### Statistical analysis

Clinicopathological features were compared among groups using the Pearson χ^2^ test. Overall survival was estimated with the Kaplan-Meier method, and differences were assessed using the log rank test. A Cox regression model was used to estimate hazard ratios of variables in the univariate analysis; significant variables were included in the multivariate Cox model. *P*-values <0.05 indicated statistical significance. Statistical analysis was performed with SPSS version 20.

## RESULTS

### Clinicopathological characteristics

Seventy-one (24.14%) out of 294 patients were 40 years old or younger, 129 (43.87%) were from 41 to 65 years old, and 94 (31.97%) were 66 years old or older at the times of gastric cancer diagnosis. There was a trend toward a higher female to male ratio in the YA group (1.08:1) than in the OA (0.61:1) and E groups (0.7:1). Among patients with available Lauren histological classification, diffuse-type tumors were predominant in the YA (90.16%) and OA groups (53.92%) and less common in the E group (29.68%); however, there were high rates of missing data in all groups (YA 14.1%; OA 20.9%; E 31.9%). Although most patients in all age groups were diagnosed at an advanced clinical stage (CS III or IV), this proportion in the YA group was significantly higher (YA 88.7%; OA 69.9%; E 67.1%; *p*=0.009). Moreover, the lowest rates of surgery (48.9%) and chemotherapy (17.2%) were observed in the E group. This data is summarized in Table 1.

### Overall survival

**Figure 2:**
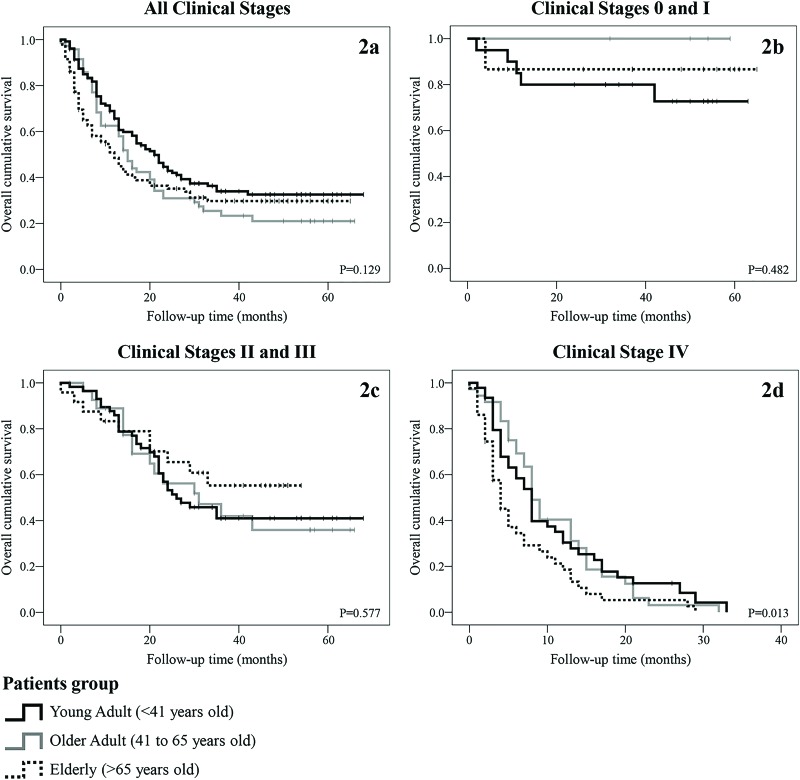
Overall survival. **(2a)** Overall survival according to patient age group. **(2b)** Overall survival in clinical stages 0 and I according to patient age group (YA N=4; OA N=20; E N=15). **(2c)** Overall survival in clinical stages II and III according to patient age group (YA N=28; OA N=57; E N=24). **(2d)** Overall survival in clinical stage IV according to patient age group (YA N=36; OA N=46; E=43).

For all 294 patients, the mean and median follow-up were 20.65 and 14 months, respectively, and the estimated mean and median overall survival were 28.58 and 16 months, respectively. For the YA, OA and E groups, the mean overall survival was 24.99, 31.80 and 25.57 months, and the median survival was 15, 21 and 12 months, respectively. There were no significant differences in overall cumulative survival among groups (*p*=0.129), but the OA group tended to have the best outcome. The two-year survival rates were 31%, 45.9%, and 35.1% in the YA, OA and E groups, respectively. Figure 2a shows the Kaplan-Meier survival curve for all clinical stages for each group.

To analyze cumulative survival according to CS, patients were divided into the early stage disease (CS 0 and I), locally advanced disease (CS II and III) and metastatic disease groups (CS IV). Figures 2b and 2c show the Kaplan-Meier survival curves for patients in the early stage and locally advanced disease groups, respectively, according to age group. There were no significant differences in survival. Figure 2d shows the Kaplan-Meier survival curve for patients in the metastatic disease group; among these patients, the E group had a significantly poorer outcome. Table 2 summarizes the cumulative 2-year survival rate for each group.

### Disease-free survival

**Figure 3:**
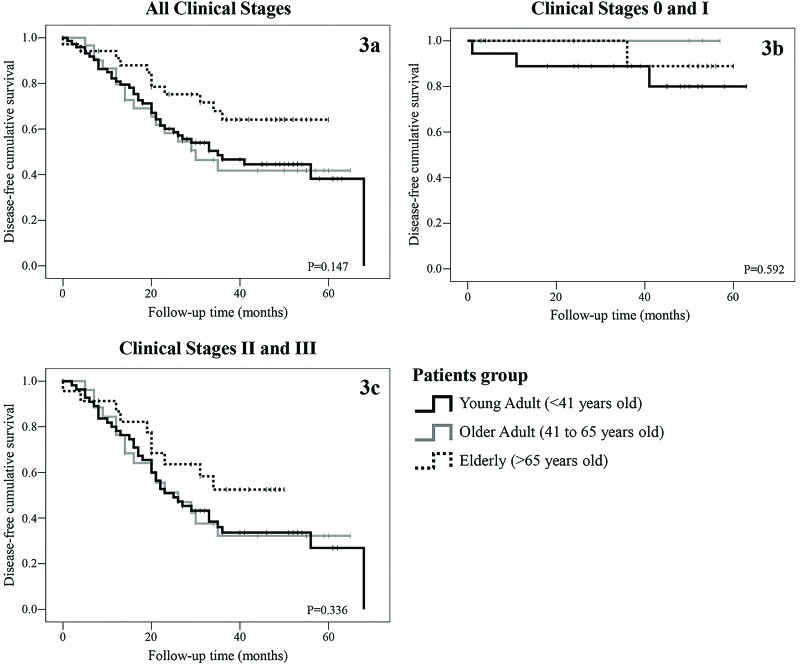
Disease-free survival. **(3a)** Disease-free survival according to patient age group. **(3b)** Disease-free survival in clinical stages 0 and I according to patient age group (YA N=4; OA N=19; E N=13). **3c.** Disease-free survival in clinical stages II and III according to patient age group (YA N=27; OA N=56; E N=23).

For the disease-free survival analysis, we included 142 patients (YA N=31; OA N=75; E N=36) who underwent curative surgery. Follow-up time was calculated from the surgery date to the date of recurrence or death. There were no significant differences in disease-free survival, although the E group tended to have a better outcome. Figure 3a-c and Table 2 summarize the results.

### Prognostic factors

In the univariate analysis with all patients, diffuse-type tumors, CS, no surgery and no chemotherapy were significantly associated with an increased hazard ratio of death. Locally advanced and metastatic didease at diagnosis and no chemotherapy remained significant in the multivariate analysis.

Prognostic factors exclusive for YAs were also evaluated. The multivariate analysis demonstrated that CS IV at diagnosis was the only factor significantly associated with an increased hazard ratio for death in this group. Table 3 summarizes these findings.

## DISCUSSION

Cancer is usually caused by cumulative genetic mutations and epigenetic alterations; therefore, it is more common in older individuals. For gastric cancer, the majority of patients are diagnosed after 60 years of age. Nevertheless, the stomach cancer incidence among younger individuals appears to be increasing 2,3, and recent studies have reported conflicting results regarding survival in this set of patients 5,6,9. To analyze whether there are differences in survival and prognostic factors between younger and older patients with gastric cancer in Brazil, this retrospective cohort study with 294 patients from the country's largest cancer center was designed.

In this study, there was a higher proportion of women among the younger patients (52.1% in YA, 38% in OA and 41.5% in E), which is in agreement with the findings of previous reports 4-9,13,18. Although the reasons for this difference are not clear, two potential explanations have been identified so far. First, men are more frequently exposed to known gastric cancer risk factors, such as smoking and alcohol intake, which might contribute to increased gastric cancer incidence later in life. Second, a decreased risk of stomach cancer was associated with longer years of fertility and postmenopausal hormone replacement therapy 25-27. As women aged 40 or younger might not have benefited from these protective factors, the incidence rates of males and females in younger groups of patients tend to equalize.

Several studies, including those performed in Brazil, found that the diffuse histological subtype is more commonly detected in younger individuals 4-9,13,18. In our study, 93.4% of the younger patients for whom detailed histological information was available presented with this subtype of cancer. Germline mutations, specifically in the CDH1 gene, associated with gastric cancer diagnosis at a younger age and with a diffuse phenotype might have contributed to this disproportion. In Brazil, a previous study of individuals with a family history of gastric cancer found CDH1 germline pathogenic mutations in 2 of 4 families 28. Nevertheless, our group did not find any pathogenic mutations in the CDH1 gene in a group of Brazilian individuals aged <55 years with gastric cancer. The pathogenesis of the intestinal subtype involves a sequence of preneoplastic lesions that take longer to develop, and therefore, this subtype is more frequently detected in older individuals.

Differences in TNM stage at diagnosis according to age group were found in this study. Younger patients more frequently presented with locally advanced and metastatic disease at diagnosis, which is in agreement with previous findings 5,29. It is possible that gastric cancer is not considered as a differential diagnosis in young patients with gastrointestinal symptoms, which could delay investigation and diagnosis. Furthermore, the findings might indicate that gastric cancer has a more aggressive course in young individuals.

Younger and older patients with gastric cancer had similar overall cumulative survival rates in our study. Nevertheless, in our study, YA had the lowest overall 2-year survival rate (31%). This finding is in agreement with those of other recent studies 8,9,30,31. However, some studies have reported worse survival rates among young patients 5,32,33, while others have found higher survival rates for younger patients 6,7,18.

Several reasons may explain this inconsistency. First, definitions of study groups are diverse. In recent studies, the cut-off age to define young patients has varied from 35 in Smith and Stabile 5 to 50 years in Pisanu et al. 34. Moreover, definitions of control groups have differed, with some studies considering two to five groups with consecutive ages and others analyzing two groups with nonconsecutive ages. Second, a small number of young patients, sometimes fewer than 50 5,32,34,35, may have contributed to the reported discrepancies. Third, regional variations in gastric cancer incidence and mortality rates might be implicated in this difference 1. In Eastern countries, most studies found no differences 9,30,31 or better survival rates 5,18 for young patients. Among Western countries, two studies reported better outcomes for this group 7,35, two found worse outcomes 5,32, and two detected no differences in survival rates for YAs 8,34. Finally, different treatment courses might have contributed to the outcome differences observed among studies.

After adjusting for CS, the differences in survival were significant among patients in the CS IV group, in which E patients had the poorest survival rate (3.2%). In our study, CS II, III and IV at diagnosis, no surgical treatment and no chemotherapy were all significantly associated with poorer prognosis in all patients. For the YA group, only metastatic disease at diagnosis was a significant prognostic factor, perhaps because practically all young patients with stage I-III disease in our sample underwent surgery (32 of 33). Additionally, there were no differences in disease-free survival, possibly due to the small number of patients diagnosed with resectable tumors, especially in the YA group.

Our study is the first to our knowledge to evaluate survival rates and prognostic factors in YAs with gastric cancer in Brazil. Our study limitations include the limited number of patients and the selection of controls. However, we provide new and clinically valuable data on a disease with increasing incidence in YAs, a group in which cancer may have considerable social and psychological impacts. In this scenario, young patients were rarely diagnosed with early stage disease, which is why they tended to have low survival rates. It remains unclear whether this is due to a diagnosis delay or more aggressive disease behavior. Therefore, awareness among YAs should be improved in order to diagnose gastric cancer at a curable stage.

There was no difference in the overall survival of YAs and OAs with gastric cancer. Nevertheless, YAs were more frequently diagnosed with metastatic disease, the only factor predictive of death in this age group. These findings suggest the need for a better approach to diagnose early gastric cancer in patients aged 40 years or younger.

## AUTHOR CONTRIBUTIONS

Cormedi MC collected and analyzed the data and wrote the manuscript. Katayama ML interpreted the results and wrote the manuscript. Guindalini RS collected the data. Faraj SF revised the pathological information. Folgueira MA designed and supervised the research, interpreted and discussed the data, and wrote and submitted the manuscript.

## Figures and Tables

**Table 1:** Clinicopathological features of gastric cancer patients according to age group.

Characteristic	Younger Adult (≤40 years) N=71	Older Adult (41 to 65 years) N=129	Elderly (>65 years) N=94	*P*-value
Age at diagnosis (years)				
Median	37	57	73	
Gender				
Female	37/71 (52.1%)	49/129 (38.0%)	39/94 (41.5%)	0.15
Histology				
Intestinal	3/61 (4.9%)	39/102 (38.2%)	39/64 (60.9%)	<0.01*
Diffuse	57/61 (90.2%)	55/102 (54.0%)	19/66 (29.7%)	
Mixed	3/61 (4.9%)	8/102 (7.8%)	6/64 (9.4%)	
Clinical stage				
0 or I	4/68 (5.9%)	20/123 (16.3%)	15/82 (18.3%)	<0.01*
II	5/68 (7.4%)	17/123 (13.8%)	12/82 (14.6%)	
III	23/68 (33.8%)	40/123 (32.5%)	12/82 (14.6%)	
IV	36/68 (52.9%)	46/123 (37.4%)	43/82 (52.4%)	
Surgery	45/70 (64.3%)	93/128 (72.7%)	43/88 (48.9%)	<0.01*
Chemotherapy	21/70 (30%)	44/124 (35.5%)	15/87 (17.2%)	0.01*

* Statistically significant

**Table 2:** Two-year cumulative survival rate (%).

	Younger Adult (≤40 years)	Older Adult (41 to 65 years)	Elderly (>65 years)
Overall survival	N=71	N=129	N=94
All	31	45.9	35.1
Clinical stage			
0 or I	100	80	86.7
II or III	56.2	53.1	65.5
IV	3.1	12.7	5.3
Disease-free survival	N=31	N=75	N=36
All	49.2	59.2	73.1
Clinical stage			
0 or I	80	90.9	90
II or III	42.9	47.5	65.5

**Table 3:** Hazard ratios of death for all patients and for younger adults.

	All patients		Younger adults	
	HR (95% CI)	*P*-value	HR (95% CI)	*P*-value
Univariate				
Age (ref: Elderly)				
Younger adult	0.94 (0.65-1.37)	0.75	-	
Older adult	0.66 (0.52-1.02)	0.06	-	
Sex (ref. male)				
Female	0.95 (0.72-1.27)	0.77	0.94 (0.54-1.63)	0.83
Histology (ref. intestinal)				
Diffuse	1.55 (1.07-2.24)	0.02	0.99 (0.24-4.11)	0.99
Mixed	1.00 (0.49-2.07)	0.98	1.79 (0.29-10.80)	0.52
Clinical stage (ref. 0 or I)			(Ref. 0, I, II or III)	
II or III	3.59 (1.63-7.90)	<0.01*	-	
IV	20.18 (9.18-44.35)	<0.01*	6.35 (3.20-12.59)	<0.01*
Surgery (ref: yes)				
No	5.53 (4.03-7.58)	<0.01*	4.16 (2.25-7. 70)	<0.01*
Chemotherapy (ref: yes)				
No	3.08 (2.13-4.47)	<0.01*	3.35 (1.71-7.34)	<0.01*
Multivariate				
Histology (ref. intestinal)				
Diffuse	1.14 (0.75-1.75)	0.52	-	
Mixed	1.63 (0.725-3.54)	0.24	-	
Clinical stage	(ref. 0 and I)		Ref. 0, I, II, and III	
II or III	13.50 (4.04-45.06)	<0.01*	-	
IV	34.75 (10.07-19.92)	<0.01*	4.84 (1.76-13.33)	<0.01*
Surgery (ref: yes)				
No	1.42 (0.86-2.34)	0.17	1.35 (0.67-2.73)	0.40
Chemotherapy (ref: yes)				
No	2.34 (1.35-4.05)	<0.01*	1.19 (0.42-3.36)	0.73

* Statistically significant
